# Interaction of hope and optimism with anxiety and depression in a specific group of cancer survivors: a preliminary study

**DOI:** 10.1186/1756-0500-4-519

**Published:** 2011-11-28

**Authors:** Rama K Rajandram, Samuel MY Ho, Nabil Samman, Natalie Chan, Colman McGrath, Roger A Zwahlen

**Affiliations:** 1 Oral & Maxillofacial Surgery, Faculty of Dentistry, The National University of Malaysia, Kuala Lumpur, Malaysia; 2 Department of Applied Social Studies, The City University of Hong Kong, Tat Chee Avenue, Kowloon Tong, Hong Kong SAR, People's Republic of China; 3 Dept of Psychology, The University of Hong Kong, Knowles Building, Kowloon Tong, Hong Kong SAR, People's Republic of China; 4 Oral & Maxillofacial Surgery, Faculty of Dentistry, The University of Hong Kong, 34,Hospital Road, Kowloon Tong, Hong Kong SAR, People's Republic of China; 5 Dept. of Public Health, Faculty of Dentistry, The University of Hong Kong, 34 Hospital Road, Kowloon Tong, Hong Kong SAR, People's Republic of China

## Abstract

**Background:**

Anxiety and depression have been identified as a common psychological distress faced by the majority of cancer patients. With the increasing number of cancer cases, increasing demands will be placed on health systems to address effective psychosocial care and therapy. The objective of this study was to assess the possible role of hope and optimism on anxiety and depression. We also wanted to investigate if there is a specific component of hope that could play a role in buffering anxiety and depression amongst cancer patients.

**Methods:**

A retrospective cross sectional study was conducted in the outpatient station of the Oral and Maxillofacial Surgery at the University of Hong Kong, Hong Kong SAR, PR-China. Fifty patients successfully treated for OC cancer were recruited after their informed consents had been obtained during the review clinic. During their regular follow-up controls in the outpatient clinic the patients compiled the hospital anxiety and depression scale (HADS), hope scale (HS) and the life orientation scale-revised (LOT-R).

**Results:**

Hope was negatively correlated with depression (*r *= -.55, *p *< .001) and anxiety (r = -.38, *p *< .05). Similar pattern was found between optimism and the latter adjustment outcomes (depression: *r *= -.55, *p *< .001; anxiety: *r *= -.35, *p *< .05). Regression analyses indentified that both hope and optimism were significant predictors of depression. Hope and optimism had equal association with depression (hope: *β *= .40 versus optimism: *β *= .38). Hope and optimism together were significantly predictive of anxiety, whereas neither hope nor optimism alone was significant individual predictors of anxiety.

**Conclusions:**

Hope and optimism both negatively correlated with patients' level of anxiety and depression. Besides theoretical implications, this study brings forward relevant findings related to developing specific clinical psychological care in the field of oncology that to date has not been researched specifically in the field of oncology. The results of this study will help guide the direction of future prospective studies in the field of oncology. This will contribute significantly to increasing patients quality of life as well enabling health care facilities to provide all cancer patients a more holistic cancer care.

## Background

It is widely accepted in current times that successful cancer treatment is no longer only determined by a cancer free state but also by its impact on psychological well-being and quality of life. Over this past two decades, the construct of hope and optimism has started to receive increasing attention in oncology. Studies have shown that hope is associated with positive features of coping with cancer, increased well being, and lower anxiety and depression symptoms [1-3]. Although hope and optimism both involve trait like thoughts about goals and are often used interchangeably, research has shown that these two constructs are distinctive and may lead to positive outcomes through entirely different mechanisms [4,5]. Hope is formally defined as a positive motivational state that comprises two components which are the agency and pathways components [5,6]. The agency component is the motivational component of hope that is used to initiate and sustain the movement toward achieving a goal [6]. The pathways component refers to the perceived ability in generating successful routes to attain the given goal [6]. Optimism is defined as a stable tendency to believe that good rather than bad things will happen [7]. In optimism, the general positive outcome expectation is the central determinant of persistent efforts in attaining the desired positive goal [5,7]. The major distinction between the two constructs is that hope emphasizes the individual motivation and perceived successful pathways that will play an integral part in developing positive outcome expectancies in comparison to optimism in which the focus is on particular actions that may bring those positive events [5]. Specifically, greater hope and optimism would positively motivate the desired attitude towards achieving a desired outcome [5]. However, there have been no studies examining the effect of hope and optimism onto anxiety and depression in cancer patients.

Oral cavity cancer (OCC) provides a good model for research of the theories of hope and optimism due to the fact that high levels of psychopathology have been found amongst patients from this cancer group. Anxiety and depression, which are two of the common psychopathology in cancer patients, have been detected to be as high as 30 to 40% in OCC patients; both being unrelated to the impact of diagnosis and/or its treatment [8]. This is because individuals with OCC face numerous challenges in dealing with this disease and its treatment modalities often lead to facial disfigurement, impaired functions of speech, eating, swallowing and chewing, as well as obvious aesthetic impairments. Ultimately, the sequelae of OCC can lead to financial, social and occupational problems [9]

To date studies in the field of oncology have primarily focused on investigating the impact of the disease and its treatment related variables regarding the adjustment to cancer; however, such issues do not always correspond or correlate with the outcome of patients' adjustment. Cancer patients have been shown to have positive as well as negative adjustments [10-13] during their encounter with this life threatening disease. There has been little effort in the field of oncology to integrate both the theories hope and optimism and its relationship with anxiety and depression in patients suffering from a life threatening disease like cancer [5,14]. Besides that, we also found that there was no studies properly investigating the specific roles of each component of hope on anxiety and depression in oncology patients [5,14].

The aim of this study was to expand the existing literature by examining the effect of hope and optimism on the two most common negative psychological adjustments in oncology: anxiety and depression. Specifically, we were interested in whether hope and optimism would be significantly related to psychological distress (i.e., anxiety and depression) after accounting for the contribution of demographic and disease characteristic variables. We also wanted to investigate if there was a specific component of the trait hope which was significantly involved in reducing anxiety and depression in this specific group of oncology patients. We hypothesized that higher levels of hope and optimism would be related to lower psychological distress. Secondly, we hypothesized that there would be a specific component of hope that influenced the interaction of hope with anxiety and depression.

## Methods

This cross-sectional study followed the principles outlined in the Declaration of Helsinki [15] and was approved by the Institutional Review Board of the University/Hospital Authority Hong Kong West Cluster (HKU/HA HKW/IRB UW 09-374). All subjects were informed about the purpose of the study and signed a consent form before participating.

### Study sample

All tumour free patients with OCC stages I to IV [16] at diagnosis without distant metastases who had received treatment at the Oral and Maxillofacial Surgery outpatient clinic of the University of Hong Kong between 2000 and 2010 (*n *= 62) were invited to participate in the current study. Among the 62 patients identified, 2 of them did not have sufficient command of Cantonese (3.2%) and were excluded from the study. Of the remaining 60 patients, 50 of them agreed to participate (response rate = 83%). Patients included in this study were included based on the following eligibility criteria: native Cantonese speakers and a minimum follow-up period of six months after tumour treatment completion and diagnosed with oral cavity cancer involving anatomical regions limited to oropharynx, gingival, floor of the mouth, tongue, salivary glands, buccal mucosa and palate. All patients had been operated between January 2000 and January 2010 for a previously untreated OC cancer with or without postoperative radiotherapy and/or concurrent chemotherapy.

### Data collection

The patients were recruited from a regular follow-up control in the outpatient station of the Oral and Maxillofacial Surgery at the University of Hong Kong. Socio-demographic information (age, gender, religion, education level, income) and disease related information (time since diagnosis, stage of disease, and treatment type) were collected. Participants were invited to self complete a questionnaire incorporating standardized assessment instruments of anxiety and depression, hope and optimism. One of the co-authors (CN) was available to answer queries from the participants.

### Data collection instruments

#### a) Depression and anxiety: hospital anxiety and depression scale (HADS)

The HADS [17] is a 14-item scale used to assess the psychological states, i.e., anxiety (7-item) and depressive symptoms (7-item), of clinical patient with physical problems, including cancer patients. Participants rated each item on a 4-point Likert scale (0 = not at all and 3 = very much indeed). Higher scores implied higher anxiety and depression level. For both anxiety and depression we have used the cut off values recommended by Zigmoid and Snaith where each person is grouped according to a clinically tested classification of psychiatric morbidity where a score of < 8 is within normal range, 8-10 indicates a possible and > 10 a probable mood disorder [17]. The internal reliability alpha values for the anxiety and depression in the current study were .84 and .75 respectively.

#### b) Trait hope: adult hope scale (AHS)

The Chinese version of the Hope Scale by Ho et al. [18] was used to assess patients trait levels of hope [19]. The scale contains four *Agency *and four *Pathway *items, which were mixed with four filler items in administration. Each item is rated on a 4-point Likert scale (1 = strongly disagree, 4 = strongly agree) in which a high score indicates a higher level of pathway and agency component. The hope scale reflects the sum of the agency and pathways items. The Hope scale evidences concurrent construct validity in terms of its correlations with other related measures and it has a discriminant utility in predicting goal related outcomes beyond variances attributable to other measures [6]. Internal reliability alpha values were calculated at 0.76, 0.70, and 0.81 respectively for the pathway subscale, agency subscale and total hope scale.

#### c) Trait optimism: the Chinese version of the life orientation scale-revised (LOT-R)

The LOT-R was used to assess the patients' trait level of optimism [20]. The Life Orientation Scale-Revised was originally developed by Scheier, Carver and Bridges [7] and its Chinese version was validated by Lai, Cheung, Lee, & Yu [21]. The scale comprises 6 items, in which three positive-worded and three negative-worded items is used to measure positive and negative outcome expectancies respectively. A 4-point Likert scale (1 = strongly disagree, 4 = strongly agree) is used to rate the patients response. The internal reliability alpha (α = 0.66) value for optimism in the current study was moderate but acceptable.

### Data analysis

Descriptive statistics of the psychological measures was produced by way of mean and standard deviation values. A Pearson correlation matrix analyses was conducted to determine the strength and statistical significance level of correlation among the psychological assessment indices. Variations in psychological status were examined with respect to socio-demographics, OCC stage and treatment modality using independent sample t-test and one way analysis of variance (ANOVA) as appropriate. To examine and compare the independent effect of hope and optimism on psychological adjustment, hierarchical multiple regression analyses were performed.

## Results

### Descriptive statistics

#### Overall well-being

Table 1 illustrates the descriptive statistics of the participant's scores in the three questionnaires, Hope Scale, Life Orientation Test-Revised, and Hospital Anxiety and Depression Scale. Socio-demographic data revealed the mean age of patients was 60 years (SD = 13.06). Majority of patients were female (58%). Mostly were married (89%), received at least elementary level of education (84%). Mean survival time was 3.6 years (SD = .34). In relation to disease characteristics, 89% were diagnosed stage I and stage II cancer [16].

**Table 1:** Demographic and medical information of participants (*n *= 50)

Variables	*N*	%
Educational level		

No formal education	8	16

Primary education	14	28

Middle school	6	12

High school	10	20

University or above	12	24

Gender		

Male	21	42

Female	29	58

Religion		

Yes	22	42

No	28	58

Marital status		

Single	6	12

Married	44	88

Income		

Below 5000	12	24

5000-10000	13	25

10000 and above	25	51

Treatments		

Surgery only	34	68

Surgery + Radiotherapy	16	32

Cancer stages		

Stage I + Stage II	41	82

Stage III + Stage IV	5	10

Missing information	4	8

Table 2 shows that the levels of anxiety and depression based on the cut off values recommended by Zigmoid and Snaith [17] in our group of oral cavity cancer patients was generally low (HADS-Anxiety: *M *= 3.8, *SD *= 2.58; HADS-Depression: *M *= 3.7, *SD *= 2.57), with only 10% and 8% of participant fell above the clinical cut-off for anxiety and depression, respectively. Additionally, OCC survivors were found to be generally hopeful and optimistic. The mean score on optimism (*M *= 14.20) was comparable to the levels reported by a study done on health population. On the other hand, levels of hope (*M *= 25.66) was noted to be even higher than those detected in healthy adults by prior studies [6,22].

**Table 2:** Mean and standard deviations of all psychological measures

Psychological measure	Mean	Standard deviation
HS	25.66	4.93

LOT-R	14.20	3.58

HADS	7.60	4.37

HADS-Depression	3.70	2.57

HADS-Anxiety	3.80	2.58

*N *= 50

*HS *hope scale, *LOT-R *life orientation test-revised, *HADS *hospital anxiety and depression scale

### Description

#### Correlation analysis of hope, optimism and adjustment outcomes

In order to examine the relations between adjustment outcomes and the coping variables in the study population, partial correlations were calculated between anxiety, depression, hope and optimism (see Table 3). Results revealed hope and optimism were positively correlated with each other at a moderate level (*r *= .44, *p *< .01). Regarding their link to adjustment outcomes, hope was found to be negatively correlated with depression (*r *= -.55, *p *< .001) and anxiety (r = -.38, *p *< .05). A similar pattern was also found between optimism and the two adjustment outcomes (depression: *r *= -.55, *p *< .001; anxiety: *r *= -.35, *p *< .05).

**Table 3:** Correlation among hope, optimism and adjustment outcomes

		HADS Depression	HADS anxiety	HS	LOT-R
HADS-Depression	-	.46(**)	-.55(***)	-.55(***)	

HADS-Anxiety		-	-.38(*)	-.35(*)	

HS			-	.44(**)	

LOT-R				-	

*n *= 50

*HS *hope scale, *LOT-R *life orientation test-revised, *HADS *hospital anxiety and depression scale

***Correlation is significant at the 0.001 level (2-tailed)

**Correlation is significant at the 0.01 level (2-tailed)

*Correlation is significant at the 0.05 level (2-tailed)

#### Effects of demographic and medical variables on psychological measures

Bivariate analyses failed to identify any significant association between anxiety and socio-demographic factors (*P *> 0.05), nor anxiety and OCC stage (*P *> 0.05) nor with anxiety and OCC treatment modality (*P *> 0.05). Likewise, no significant association between depression and socio-demographic factors (*P *> 0.05), nor depression and OCC stage (*P *> 0.05) nor depression with OCC treatment modality (*P *> 0.05) were observed.

#### Hope and optimism predicting depression and anxiety

Hope and optimism were observed to be negatively and significantly correlated with depression and anxiety. To further examine and compare the independent effect of hope and optimism on psychological adjustment, two hierarchical multiple regression analyses were conducted. Income was controlled in all these analyses and was entered into step 1. Hope and optimism was then entered simultaneously into step 2, whereas depression and anxiety served as criteria. Results of these regression analyses are shown in Table 4.

**Table 4:** Optimism and hope predicting psychological adjustments

Variable	*Β*	*SE B*	*Beta*	*t*
Depression				

Hope	-.21	.07	-.40**	-3.10

Optimism	-.27	.09	-.38**	-3.09

Anxiety				

Hope	-.15	.08	-.29	-1.87

Optimism	-.16	.11	-.23	-1.55

*Finding is significant at the 0.05 level (2-tailed)

**Finding is significant at the 0.01 level (2-tailed)

#### Hope and optimism predicting depression

Hope and optimism together accounted for 41% incremental variance to the prediction of depression, controlling for the effect of income. The test of R^2 ^change was significant (*Fchange *(2, 46) = 17.16, *p *< .001), suggesting hope and optimism to be significant predictors of depression. Hope and optimism had equal association with depression (hope: *β *= .40 versus optimism: *β *= .38). Both hope and optimism were significant independent individual predictors of depression.

#### Hope and optimism predicting anxiety

Hope and optimism accounted for 19% incremental variance to anxiety. The test of R^2 ^change was significant (F change (2, 46) = 5.28, *p *< .01), suggesting hope and optimism together to be significantly predictive of anxiety however unlike depression, neither hope nor optimism were significant independent individual predictors of anxiety.

#### Agency and pathway as unique component of hope

From the above analysis, hope was found have significant independent effect on depression. However, it remained uncertain whether the effect of hope on depression was a function largely due to the agency or pathways component, or due to equal contribution of both components of hope. Thus, two further hierarchical regression analyses were conducted. Income and optimism was entered into the step 1 as covariate. Agency and pathway were entered simultaneously into the step 2. In this way, their independent effect on depression could be investigated. Results were shown in Table 5. Findings suggested that agency had a stronger association with depression (agency: *β *= .29 VS pathways: *β *= .18). It was noted only the Agency component of hope was a significant individual predictor of depression.

**Table 5:** Agency and pathway predicting depression

Variables	*Β*	*SE β*	*Beta*	*t*
Depression				

Agency	-.27	.13	-.29*	-2.21

Pathway	-.15	.11	-.18	-1.32

*Finding is significant at the 0.05 level (2-tailed)

## Discussion

To the author's knowledge this is the first study to report the importance of both hope and optimism onto anxiety and depression in cancer patients. The homogenous group of cancer patients selected in this study as well as the known existence of high levels of anxiety and depression [8] in this group of patients provides a suitable platform to investigate this relationship and determine the direction for future prospective studies. This is the first study examining the interaction between the two dimensions of hope (i.e. pathway and agency) with anxiety and depression. Consistent with our first hypothesis, hope and optimism was both found to be negatively correlated with patients' level of anxiety and depression.

Our patients had high levels of hope and optimism which could be the reason behind the low levels of anxiety and depression detected in this study population. In fact, the score on optimism (*M *= 14.20, *SD *= 3.58) was comparable to levels reported by healthy adults [7]. Furthermore, the levels of hope in our study sample (*M *= 25.66, *SD *= 4.93) was found to be even higher than healthy adults reported by prior studies [19]. This is in agreement with previous studies done in other mixed cancer populations which found that higher levels of hope were associated with lower levels of depression [23,24]. Besides that, these findings on hope are also in accordance with other studies that reported cancer patients tend to report relatively high levels of hope [2,25]. Another possible contribution for the low levels of anxiety and depression in this study could be due to the majority of our patients being females. This supports the general finding in the psychology literature which clearly states that females tend to engaged in more positive coping methods [26,27] in comparison to males. However, due to the cross sectional design of this study, it is unclear whether it is hope and optimism causing patients to have lower levels of anxiety and depression or if patients with lower levels of anxiety and depression are more likely to have higher levels of hope and optimism.

In our study, we found that both hope and optimism accounted for a moderate proportion of variance to depression (41%) and anxiety (19%). This supports other studies on this in other fields of oncology, suggesting that a similar adjustment process may be occurring among OCC survivors as well [28,29]. Our findings also support the fact that both hope and optimism are at best only moderately correlated with each other (*r *= 0.45). This indicates that both the constructs are related but not redundant therefore potentially exerting differential effects on psychological adjustments [22,30,31].

Hope and optimism when assessed together showed significant independent effect on depression and equal association with depression. These findings leads us to believe that when trying to cope with depression both positive expectancies regarding one's own action (hope) and positive expectancies regarding the external environment outside one's control (optimism) are important to successfully overcome it.

With regards to anxiety, hope and optimism were significant predictors when taken together however both of these constructs were found to not significant individual predictors. There are two possible reasons for these results. Firstly, it could be the that hope and optimism simply have a weak association with anxiety individually. Another possibility is that both hope and optimism have noteworthy effects, but that they are both accounting for the same variance, instead of each making unique, independent contribution to the relationship with anxiety. This occurrence is often due to, at least partly, to the two variables being correlated, which is certainly the case for the hope scale and LOT-R in the current study (*r *= .45).

Our findings on hope showed that only the agency component of hope specifically predicted depression. This supports our second hypothesis in which we believed that there was a specific component of hope that interacted with psychopathology. This finding is in line with prior studies in college samples suggesting that goal directed energy (i.e., agency), rather than planning to meet goals (i.e., pathways), is a more important contributing factor to depression [22,32]. This fits in with the phenomenological description of depressive symptoms as being a significant reduction in goal directed energy. Taken together, these findings support Snyder and colleague's hope theory [6] by demonstrating that agency and pathways are distinct construct and each is an important factor in relating to different adjustment outcomes.

This study brings forward relevant findings on hope and optimism in the field of psycho oncology. Our study brings forward the role of hope, as conceptualized by Snyder et al. [6], and optimism, as conceptualized by Scheier and Carver [7], as being two important constructs needing to be highlighted in patients suffering from cancer. Our findings further contribute to the validation of Synder's model of hope which states that hope consists of two theoretical components (i.e. agency and pathways) which are related but not redundant constructs.

Besides theoretical implications, this study brings forward the future direction for psychotherapy intervention which can be used for OCC patients as well as any other fields of oncology. Previous studies have proven that hope can be enhanced through psychosocial interventions [33]. Based on our findings that bring forward the significant role of the agency component of hope, we suggest that psychotherapy programmes should be specifically directed to focus on this motivational component of hope. This will help increase patients perceived capacity to initiate and sustain movement along a pathway until the positive goal is reached. Our current findings would support implementation of the psychosocial intervention module proposed by Berendes et al. [14] in their paper. The important components of the module would include 1) detail discussion on improving patients understanding of the disease that they are facing, 2) help patients create goals and create an ordering of important goals, 3) identify realistic short and long-term goals that are achievable within the context of oral cavity cancer, 4) identification of all the potential pathways which can lead to achieving the goals and selecting the pathway with the highest chance of achieving the determined goal, 5) helping patients find ways to increase agency and monitor their pathway to the goal [14]. The role of optimism can be enhanced in psycho-therapy programmes by introducing programmes that focus on active, problem-focused coping and less use of avoidance and goal disengagement coping methods [5,34]. Besides that, psychotherapy programmes should also promote optimism by increasing positive situation specific thoughts [5]. Therapy should be directed to increase generalized expectancy optimism thus making the patient expect more positive outcomes in any given goal pursuit. Increased positive expectancy will give rise to increase use of positive set of emotions and this will enable the patient to actively engage in goal pursuits even when situation become too difficult and stressful [5]. The findings of this study indicate that one complete psychotherapy programme consisting of all the components mentioned above should be implemented concurrently with the curative therapy prescribed to oncology patients in order to provide a more holistic therapy.

A few limitations of this study have to be considered. Firstly, its cross-sectional design prevents development of causal relationship between positive coping processes and anxiety and depression. However, this study design allows gathering important clinical information in a short period facilitating thereafter the design of future prospective studies. The sample size of this study is rather limited; however it has to be taken into consideration that the results obtained revealed significant association with anxiety and depression, indicating that the sample size had adequate sample power.

## Conclusions

In summary, the current study offers an insight into the potential relevance of hope and optimism in relation to anxiety and depression. It is hoped that this study will provide an impetus and direction for future prospective research related to positive coping strategies and psychopathology in OC cancer as well as other fields of oncology patients so as to support or refute the role of psychological interventional therapies in oncology care.

## Competing interests

The authors declare that they have no competing interests.

## Authors' contributions

RKR: Guarantor of integrity of study, study concept, study design, manuscript preparation. SMYH: Definition of intellectual content, data acquisition, data analysis, statistical analysis. NS: Guarantor of integrity of study, study concept, study design, manuscript preparation, clinical data acquisition. NC: Data acquisition, data analysis, statistical analysis, clinical data. CMcG: Guarantor of integrity of study, study concept, study design, manuscript preparation and editing. RAZ: Guarantor of integrity of study, study concept, study design, manuscript preparation and editing. All authors read and approved the final manuscript.
